# Venous Thromboembolism in Hematologic Malignancies: Incidence, Risk Factors, and the Role of Direct Oral Anticoagulants

**DOI:** 10.1111/ejh.70262

**Published:** 2026-08-03

**Authors:** Mario Biglietto, Silvia Sorella, Erminia Baldacci, Massimo Breccia, Sabina Chiaretti, Marcello Capriata, Livia Donzelli, Gianna Maria D'Elia, Salvatore Giacomo Morano, Ilaria Del Giudice, Maurizio Martelli, Cristina Santoro, Antonio Chistolini

**Affiliations:** ^1^ Department of Translational and Precision Medicine Sapienza University of Rome Rome Italy

**Keywords:** anticoagulation treatment, cancer associated thrombosis, hematological malignancy, oral anticoagulant, thrombotic risk, venous thromboembolism

## Abstract

Thrombotic events, particularly venous thromboembolism (VTE), are a significant source of morbidity and mortality among patients with hematologic malignancies. These patients face unique challenges due to treatment‐related complications such as thrombocytopenia, coagulopathy, and heightened bleeding risk. Traditionally managed with low molecular weight heparin (LMWH), the advent of direct oral anticoagulants (DOACs) has introduced new opportunities for both prophylaxis and treatment of cancer‐associated thrombosis (CAT). This narrative review explores the disease‐specific thrombotic risk across hematologic malignancies and critically evaluates the current evidence supporting the use of DOACs in this setting. While emerging data suggest DOACs are effective and safe for VTE treatment and secondary prophylaxis, although with a higher incidence of clinically relevant non‐major bleeding in some studies, their role in primary prophylaxis remains under investigation and is currently limited by insufficient disease‐specific data. Laboratory monitoring may be warranted in the presence of anti‐cancer treatment‐DOACs interactions or unexpected clinical events. The utility of DOACs is particularly constrained in thrombocytopenic patients, where bleeding risks are heightened and evidence remains limited. Overall, this review highlights the need for robust, prospective, disease‐specific trials to define optimal anticoagulation strategies in oncohematologic patients and refine clinical decision‐making in this complex and high‐risk population.

## 1 Introduction

Thrombotic events are a frequent complication in cancer patients, with venous thromboembolism (VTE) being prevalent and potentially life‐threatening. Cancer patients face a ninefold increased risk of VTE compared to the general population. In the oncologic setting, VTE contributes significantly to morbidity, treatment delays, and elevated mortality rates. The risk of thrombosis varies considerably depending on tumor type, disease stage, and treatment modality [1].

For over 15 years, low molecular weight heparin (LMWH) has been the mainstay of treatment for cancer‐associated thrombosis (CAT). However, the emergence of direct oral anticoagulants (DOACs) has introduced new possibilities for both prevention and treatment, offering improved convenience and potentially broader clinical utility [2, 3, 4, 5, 6, 7, 8, 9].

Anticoagulation management becomes particularly complex in patients with hematologic malignancies. These individuals often present with unique challenges, such as treatment‐induced thrombocytopenia, coagulopathy, and higher susceptibility to bleeding, all of which complicate the risk–benefit assessment for anticoagulant use [10].

This review aims to provide a comprehensive and disease‐specific overview of thrombotic risk in hematologic malignancies, with a particular emphasis on the emerging role of DOACs in both prophylactic and therapeutic settings. By examining current evidence and limitations, we seek to clarify the clinical utility of DOACs in this high‐risk and understudied population.

## 2 Methods

A narrative review was conducted to explore thrombotic risk and the use of DOACs in patients with hematologic malignancies. PubMed was selected as the source for literature retrieval as it provides extensive coverage of biomedical and clinical research. However, the exclusion of other databases, such as Embase and the Cochrane Library, may have resulted in the omission of potentially relevant studies and should be considered a limitation of this review. The literature search was performed in the PubMed database for articles published between January 2005, corresponding to the early clinical development of DOACs, and 31 March 2026. The search strategy combined medical subject headings (MeSH) and free‐text terms, including terms related to VTE, thrombosis, CAT, catheter‐related thrombosis, DOACs, and hematologic malignancies. Boolean operators were used to combine search terms. The following search strategy was applied: (“Venous thromboembolism” OR “Thrombosis” OR “Cancer‐associated thrombosis” OR “Catheter‐Related Thrombosis”) AND (“Direct oral anticoagulants” OR “DOAC” OR “Apixaban” OR “Edoxaban” OR “Rivaroxaban” OR “Dabigatran”) AND (“Hematologic Malignancies” OR “Myeloproliferative Neoplasms” OR “Myelodysplastic Syndromes” OR “Chronic Myeloid Leukemia” OR “Chronic Lymphoid Leukemia” OR “Acute Myeloid Leukemia” OR “Acute Lymphoid Leukemia” OR “Multiple Myeloma” OR “Hodgkin Lymphoma” OR “Non‐Hodgkin Lymphoma”).

Duplicate records were removed before screening. Titles and abstracts were independently screened by M.B. and A.C. for relevance to the topic. Discrepancies regarding study eligibility were resolved through discussion and consensus among the authors. Potentially eligible articles underwent full‐text review. Original articles, clinical studies, reviews, and guideline papers published in peer‐reviewed journals were considered for inclusion.

The search was restricted to articles published in English, introducing a potential language bias.

## 3 Incidence and Disease‐Specific Risk Factors for Thrombotic Events

The development of CAT is fundamentally driven by the three elements of Virchow's triad: endothelial injury, blood stasis, and hypercoagulability. Endothelial injury may arise from the malignancy itself, its direct interaction with the vascular endothelium, or as a consequence of anticancer therapies and the presence of central venous access devices (CVADs). Blood stasis is often exacerbated by prolonged immobilization, surgical interventions, and the mechanical compression of blood vessels by tumor masses. Hypercoagulability, the central feature of CAT, is predominantly mediated by the release of procoagulant, proinflammatory, and angiogenic factors from tumor cells [11].

Risk factors for thrombosis in the general population also play a role in patients with hematological malignancies and may act synergistically with cancer‐related mechanisms. These include advanced age, a personal and/or family history of VTE, as well as other established risk factors such as comorbidities and reduced mobility, which may further amplify the prothrombotic state in this population [11].

Figure 1 schematically illustrates the mechanisms contributing to hypercoagulability in malignancies.

**FIGURE 1:**
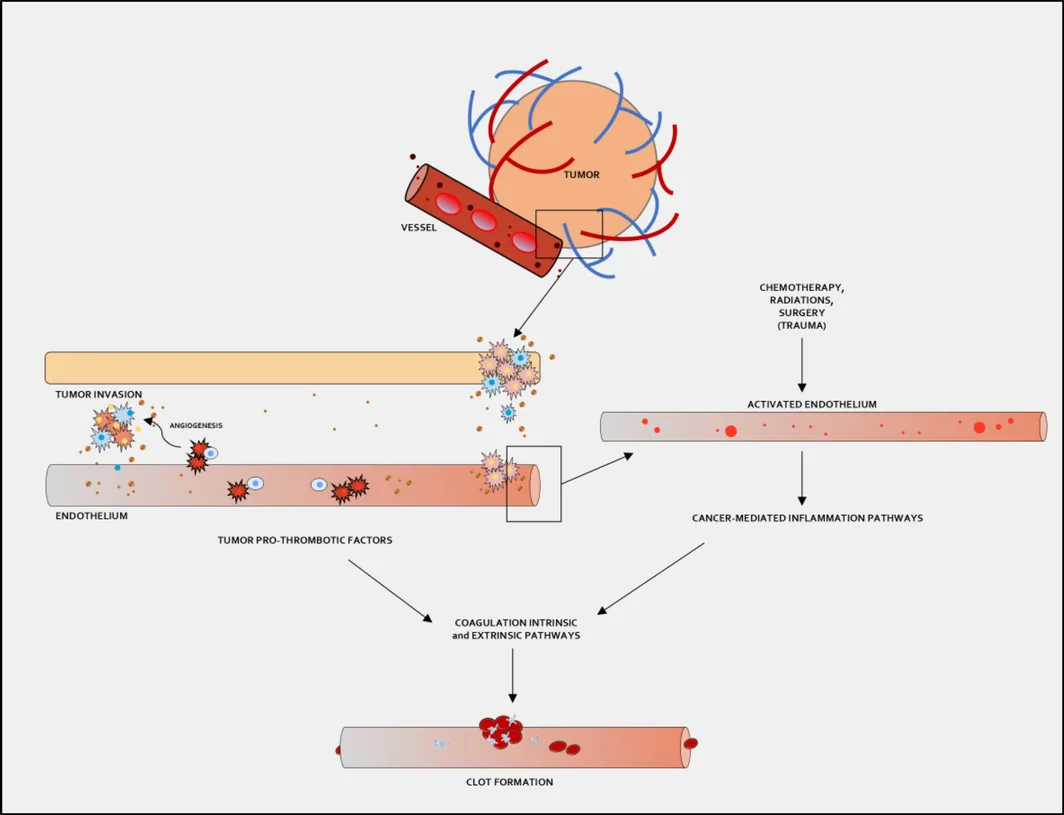
Pathogenesis of cancer‐associated thrombosis (CAT).

The following sections provide an overview of the main disease‐specific risk factors for thrombotic complications across different hematologic malignancies. While this review primarily focuses on VTE, arterial events are discussed where they represent a major component of disease‐specific thrombotic burden. A summary of these risk factors is presented in Tables 1 and 2.

**TABLE 1:** Risk factors for thrombosis and risk scores in hematologic malignancies.

Hematologic malignancy	Risk factors for thrombosis		Scores for thrombotic risk assessment
Ph‐negative MPNs	Age ≥ 60 years, history of thrombosis, cardiovascular risk factors, erythrocytosis, leukocytosis, thrombocytosis, JAK2 V617F allele burden, wt‐CALR, high‐risk mutations (BCOR/BCORL1, ASXL1, DNMT3A), increased inflammatory markers	PV	MFPS‐PV [12]
		ET Pre‐PMF	IPSET‐Thrombosis [13] Revised IPSET‐Thrombosis [14]
		PMF	No validated score
CML	Age, cardiovascular risk factors, TKI treatment		No validated score
MDS	Age, comorbidities		No validated score
MM	Immunomodulatory drugs, dexamethasone, multi‐agent chemotherapy and anthracyclines treatment, increased BMI, pathological fractures (pelvis, hip, or femur), EPO use, history of VTE, age ≥ 80 years, recent surgery (within 90 days), tunneled CVC, lack of thromboprophylaxis, abnormal metaphase cytogenetics, Asian ethnicity		IMWG risk assessment model [15] IMPEDE‐VTE [16] SAVED [17] PRISM [18]
HL	Treatment, hemoglobin, leukocyte and platelet count, BMI, previous VTE, reduced mobility, history of acute myocardial infarction or stroke, extranodal disease, mediastinal involvement, bulky disease, ECOG performance status ≥ 2, presence of a PICC		ThroLy [19] Thro‐HL [20] Khorana score [21]
NHL	Type of lymphoma, treatment, hemoglobin, leukocyte and platelet count, BMI, previous VTE, reduced mobility, history of acute myocardial infarction or stroke, extranodal disease, mediastinal involvement, bulky disease, ECOG performance status ≥ 2, presence of a PICC		ThroLy [19]Khorana score [21]
AL	Procoagulant properties of leukemic cells, patient‐specific characteristics, leukemia‐directed treatments, use of CVCs, septic complications		No validated score

Abbreviations: AL, acute leukemias; BMI, body mass index; CML, chronic myeloid leukemia; CVC, central venous catheter; ECOG, Eastern Cooperative Oncology Group; EPO, erythropoiesis‐stimulating agents; ET, essential thrombocythemia; HL, Hodgkin lymphoma; IMWG, International myeloma working group; IPSET, International Prognostic Score for Thrombosis in Essential Thrombocythemia; MDS, Myelodysplastic Neoplasms; MFPS‐PV, Mutation‐Enhanced Prognostic Scoring System for PV; MM, multiple myeloma; NHL, Non Hodgkin lymphomas; Ph‐negative MPNs, Philadelphia‐negative myeloproliferative neoplasms; PICC, peripherally inserted central catheter; PMF, primary myelofibrosis; pre‐PMF, prefibrotic primary myelofibrosis; PV, polycythemia vera; Thro‐HL, thrombosis Hodgkin lymphoma; ThroLy, thrombosis lymphoma; VTE, venous thromboembolism; wt‐CALR, CALR wild‐type.

**TABLE 2:** Scoring systems for thrombotic risk assessment in patients with hematologic malignancies.

Score	Hematologic malignancy	Score items
MFPS‐PV [12]	PV	Age > 60 years, cardiovascular risk factors, prior thrombotic events, presence of high risk mutations (ASXL1, DNMT3A, BCOR/BCORL1)
IPSET‐Thrombosis [13]	ET	Age > 60 years, prior thrombosis, cardiovascular risk factors, JAK2 V617F mutation
Revised IPSET‐Thrombosis [14]	Pre‐PMF	Age > 60 years, prior thrombosis, JAK2 V617F mutation
IMWG risk assessment model [15]	MM	BMI ≥ 30, previous VTE, CVC or pacemaker, cardiac disease, chronic renal disease, diabetes, acute infection, immobilization, blood clotting disorders, general surgery, any anesthesia, trauma, EPO, MM diagnosis, hyperviscosity, high dose dexamethasone (≥ 480 mg/month), doxorubicin
IMPEDE‐VTE [16]	MM	Risk factors: IMIDs use, BMI ≥ 25, pelvic, hip or femur fracture, EPO, doxorubicin and dexamethasone use (≤ 160 mg/month or > 160 mg/month), previous VTE, tunneled line/CVC Protective factors: Asian/Pacific Islander ethnicity, existing thromboprophylaxis (therapeutic LMWH or warfarin/prophylactic LMWH or aspirin)
SAVED [17]	MM	Risk factors: surgery (within 90 days), previous VTE, age ≥ 80 years, dexamethasone use (> 160 mg/cycle or 120–160 mg/cycle) Protective factor: Asian ethnicity
PRISM [18]	MM	Previous VTE, Black ethnicity, IMIDs use, surgery (within 90 days), abnormal metaphase cytogenetics
Thro‐HL [20]	HL	Bulky disease, ECOG PS 2–4, presence of PICC, mediastinal involvement
ThroLy [19]	HL/NHL	Previous VTE, reduced mobility, previous acute myocardial infarction or stroke, BMI ≥ 30, extranodal localization, mediastinal involvement, neutrophils < 1 × 10^9^/mmc, hemoglobin < 10 g/dL
Khorana score [21]	HL/NHL	Cancer type, pre‐chemotherapy platelet count ≥ 350 × 10^9^/mmc, hemoglobin < 10 g/dL or EPO use, pre‐chemotherapy leukocyte count > 11 × 10^9^/mmc, BMI ≥ 35

Abbreviations: BMI, body mass index; CVC, central venous catheter; ECOG PS, Eastern Cooperative Oncology Group performance status; EPO, erythropoiesis‐stimulating agents; ET, essential thrombocythemia; HL, Hodgkin Lymphoma; IMIDs, immunomodulatory drugs; IMWG, international myeloma working group; IPSET, International Prognostic Score for Thrombosis in Essential Thrombocythemia; LMWH, low molecular weight heparin; MFPS‐PV, Mutation‐Enhanced Prognostic Scoring System for PV; MM, multiple myeloma; NHL, Non Hodgkin lymphomas; PICC, peripherally inserted central catheter; Pre‐PMF, prefibrotic primary myelofibrosis; PV, polycythemia vera; Thro‐HL, thrombosis Hodgkin lymphoma; ThroLy, thrombosis lymphoma; VTE, venous thromboembolism.

### 3.1 Philadelphia‐Negative Myeloproliferative Neoplasms

#### 3.1.1 Epidemiology

Arterial and venous thrombotic events are a well‐recognized complication in Philadelphia‐negative myeloproliferative neoplasms (Ph‐negative MPNs), with variable prevalence depending on the disease subtype. The reported frequency of thrombosis occurring at or before diagnosis is approximately 34%–38.6% in polycythemia vera (PV), 9.7%–29.4% in essential thrombocythemia (ET), and 7%–13% in primary myelofibrosis (PMF). Following diagnosis, the estimated annual incidence of thrombotic events per 100 patient‐years is approximately 2.6 in PV, 1.7 in ET, 1.9 in prefibrotic PMF (pre‐PMF), 1.6 in PMF, and 2.3 in post‐PV/post‐ET myelofibrosis [22].

#### 3.1.2 Patient‐Related Risk Factors

They include advanced age (> 60 years), a prior personal history of thrombosis and traditional cardiovascular risk factors, such as hypertension, diabetes mellitus, dyslipidemia, and smoking.

#### 3.1.3 Disease‐Related Risk Factors

They include the presence of JAK2 V617F mutation, particularly with high allelic burden, as well as erythrocytosis, leukocytosis, thrombocytosis and elevated inflammatory markers (e.g., increased neutrophil‐to‐lymphocyte ratio), all contributing to endothelial activation and a procoagulant state.

#### 3.1.4 Treatment‐Related Risk Factors

Treatment‐related risk factors also influence predisposition to thrombosis. In particular, suboptimal cytoreductive therapy and inadequate hematocrit control in PV are associated with increased thrombotic risk, whereas appropriate cytoreduction and antiplatelet therapy mitigate this risk [12, 13, 14, 22, 23, 24].

#### 3.1.5 Risk Assessment Models (RAMs)

Historically, thrombotic risk stratification in PV has relied on a binary model based on age and prior thrombotic history. However, this simplified approach may be insufficient, prompting the development of more refined prognostic tools [12, 23]. The Mutation‐Enhanced Prognostic Scoring System for PV (MFPS‐PV) integrates clinical and molecular variables, including age > 60 years, cardiovascular risk factors, prior thrombosis and high‐risk somatic mutations (ASXL1, DNMT3A, BCOR, BCORL1), and has demonstrated improved predictive accuracy [12]. In ET, risk assessment is typically performed using the revised IPSET‐thrombosis score, which incorporates age > 60 years, JAK2 V617F mutation status, prior thrombosis and cardiovascular risk factors [13, 14]. In contrast, no validated scoring system currently exists for PMF, and the application of PV‐ or ET‐based models has shown limited prognostic value, although some utility has been reported in pre‐PMF [24].

### 3.2 Chronic Myeloid Leukemia

#### 3.2.1 Epidemiology

Patients with chronic myeloid leukemia (CML) have an increased risk of both venous and arterial thrombotic events, with reported incidences of approximately 11%–13% for VTE and 5%–15% for arterial events [25, 26]. Notably, arterial complications represent a major component of the thrombotic burden in this setting.

#### 3.2.2 Patient‐Related Risk Factors

They include advanced age and cardiovascular comorbidities.

#### 3.2.3 Disease‐Related Risk Factors

Disease‐related risk factors for VTE remain incompletely understood in CML.

#### 3.2.4 Treatment‐Related Risk Factors

They play a central role in thrombosis in CML. Tyrosine kinase inhibitors (TKIs), particularly second‐ and third‐generation agents, are associated with an increased incidence of arterial occlusive events (AOEs). Second‐ and third‐generation TKIs have been associated with a higher risk of arterial occlusive events compared with first‐generation imatinib. Reported effect estimates vary across studies, with increased risks observed for nilotinib, dasatinib, and ponatinib relative to imatinib [27]. Dose optimization strategies, such as reducing ponatinib from 45 mg to 15 mg after achieving a complete cytogenetic response (≥MR2), may mitigate this risk [28]. Newer TKIs, including bosutinib and the STAMP inhibitor asciminib, appear to have a more favorable cardiovascular safety profile, although long‐term data are still limited [26].

#### 3.2.5 RAMs

Currently, there is no reliable and validated risk score available to stratify patients with CML according to their risk of thrombosis.

### 3.3 Myelodysplastic Neoplasms

#### 3.3.1 Epidemiology

Myelodysplastic neoplasms (MDS) are primarily characterized by an increased risk of bleeding, while thrombotic events are less common and less well defined in this setting.

#### 3.3.2 Patient‐Related Risk Factors

They include advanced age and comorbidities, which represent the main contributors to thrombotic risk in this population [29].

#### 3.3.3 Disease‐Related Risk Factors

They appear to play a limited role in thrombosis. However, features such as systemic inflammation and clonal hematopoiesis may contribute to a prothrombotic state; however, their clinical impact remains uncertain [30].

#### 3.3.4 Treatment‐Related Risk Factors

They may be relevant in selected subgroups. In particular, lenalidomide has been associated with an increased risk of thrombosis, especially in patients with del(5q). However, available data are inconsistent. A recent study reported a thrombotic event rate of approximately 8% in patients with MDS and 5q deletion treated with lenalidomide [29, 31]. Currently, no established guidelines recommend routine thromboprophylaxis in this setting.

#### 3.3.5 RAMs

There is no reliable and validated risk score available to stratify patients with MDS according to their risk of thrombosis.

### 3.4 Multiple Myeloma

#### 3.4.1 Epidemiology

Patients with multiple myeloma (MM) have an estimated risk of VTE exceeding 10% over the course of their disease, with older studies reporting rates as high as 26% [32].

#### 3.4.2 Patient‐Related Risk Factors

They include advanced age, a history of VTE, and comorbidities. Race also plays a role, with Caucasian patients being at higher risk than Asians and Pacific Islanders [32].

#### 3.4.3 Disease‐Related Risk Factors

They also contribute to thrombotic risk in MM, reflecting a tumor‐driven hypercoagulable state characterized by inflammation, endothelial activation, monoclonal protein effects (hyperviscosity), and impaired fibrinolysis [33].

#### 3.4.4 Treatment‐Related Risk Factors

They are particularly significant in MM. The use of anthracyclines, dexamethasone, erythropoiesis‐stimulating agents, and immunomodulatory drugs (IMiDs), such as thalidomide, lenalidomide, and pomalidomide, is associated with an increased incidence of VTE. While monotherapy with IMiDs modestly contributes to baseline VTE risk (approximately 3%–4%), the risk can rise substantially, up to 26%, when combined with dexamethasone, multi‐agent chemotherapy, or anthracyclines. Other biological treatments generally have a limited impact on VTE risk [32, 33, 34].

#### 3.4.5 RAMs

Since the Khorana score does not adequately predict thrombotic events in MM, several MM‐specific risk models have been developed [35]. The International Myeloma Working Group (IMWG) model incorporates patient‐, disease‐, and treatment‐related variables [15]. More recently, the IMPEDE‐VTE, SAVED, and PRISM scores have been introduced. The IMPEDE‐VTE score includes factors such as IMiD therapy, body mass index, pathological fractures, use of erythropoiesis‐stimulating agents, dexamethasone or doxorubicin, ethnicity, history of VTE, presence of a central venous catheter (CVC), and thromboprophylaxis [16]. The SAVED score incorporates prior surgery, Asian race, history of VTE, age ≥ 80 years, and dexamethasone dose [17]. The PRISM score includes prior VTE, race, IMiD treatment, recent surgery, and cytogenetic features [18]. Among these, the IMPEDE‐VTE score appears to have the best performance in real‐world settings, although its superiority has not been definitively established [36].

### 3.5 Hodgkin Lymphoma

#### 3.5.1 Epidemiology

The incidence of thrombosis in patients with Hodgkin Lymphoma (HL) has been reported to range between 3% and 12% [19, 20, 37].

#### 3.5.2 Patient‐Related Risk Factors

They include advanced age and reduced mobility, which may contribute to an increased baseline thrombotic risk.

#### 3.5.3 Disease‐Related Risk Factors

They play a relevant role in thrombotic risk in HL. In particular, bulky disease and mediastinal involvement may contribute to venous stasis and vascular compression, thereby promoting thrombus formation. In addition, systemic inflammation and high tumor burden may further enhance the prothrombotic state.

#### 3.5.4 Treatment‐Related Risk Factors

Treatment‐related factors may also contribute to thrombotic risk. The use of CVCs, particularly peripherally inserted central catheters (PICCs), represents a major risk factor in this setting. In addition, chemotherapy‐related endothelial injury and the use of corticosteroids may further increase the risk of thrombosis.

#### 3.5.5 RAMs

Historically, thrombotic risk assessment in this population has relied on the Khorana [21] and Thrombosis Lymphoma (ThroLy) scores [19]. The Khorana score estimates thrombotic risk based on cancer type, pre‐chemotherapy platelet count, hemoglobin concentration, leukocyte count, and body mass index (BMI) [21]. The ThroLy score incorporates a broader range of clinical variables, including prior VTE, reduced mobility, history of acute myocardial infarction or stroke, BMI, extranodal disease, mediastinal involvement, neutropenia, and hemoglobin levels [19]. However, both scores have demonstrated suboptimal performance in accurately stratifying thrombotic risk among patients with HL [20]. In response to this limitation, a novel prognostic model, the Thrombosis Hodgkin Lymphoma (Thro‐HL) score, was recently developed specifically for patients with classical HL [20]. This model is based on four clinical risk factors: bulky disease, an Eastern Cooperative Oncology Group performance status (ECOG PS) ≥ 2, the presence of a PICC, and mediastinal involvement. However, validation in larger prospective cohorts is required to confirm its clinical utility.

### 3.6 Non‐Hodgkin Lymphomas

#### 3.6.1 Epidemiology

Non‐Hodgkin lymphomas (NHLs) represent a heterogeneous group of malignancies, encompassing both aggressive and indolent subtypes. The overall incidence of thrombosis among patients with NHL is approximately 6%–7% [37, 38].

#### 3.6.2 Patient‐Related Risk Factors

They include advanced age and reduced mobility, which may contribute to an increased baseline thrombotic risk.

#### 3.6.3 Disease‐Related Risk Factors

They play a relevant role in thrombotic risk in NHLs as in HL. Aggressive forms of NHL present a higher risk for thrombotic events compared to those with indolent subtypes [37, 38].

#### 3.6.4 Treatment‐Related Risk Factors

Treatment‐related factors may also contribute to thrombotic risk. The use of CVCs, particularly PICCs, represents a major risk factor in this setting. In addition, chemotherapy‐related endothelial injury and the use of corticosteroids may further increase the risk of thrombosis.

#### 3.6.5 RAMs

Similar to HL, thrombotic risk in NHLs has been assessed using the Khorana and ThroLy scores; however, their predictive performance has been suboptimal in this context too [19, 20, 21], highlighting the need for reliable disease‐specific risk assessment tools.

### 3.7 Acute Leukemias

#### 3.7.1 Epidemiology

The reported incidence of thrombosis in patients with acute leukemias (AL) varies widely, ranging from 2% to 25%, depending on leukemia subtype and patient characteristics. VTE is consistently more frequent than arterial events; however, both may occur, particularly in acute promyelocytic leukemia (APL), which carries the highest thrombotic risk among AL subtypes [39, 40, 41, 42].

#### 3.7.2 Patient‐Related Risk Factors

They include advanced age, comorbidities, and reduced performance status, which may contribute to an increased baseline thrombotic risk.

#### 3.7.3 Disease‐Related Risk Factors

Disease‐related factors contribute to thrombotic risk in AL. High leukocyte count and tumor burden may promote hypercoagulability and vascular complications. This is particularly evident in APL, where a complex coagulopathy characterized by both bleeding and thrombosis is frequently observed. In addition, leukemia‐related systemic inflammation and endothelial activation may further contribute to thrombus formation.

#### 3.7.4 Treatment‐Related Risk Factors

Among leukemia‐specific therapies, L‐asparaginase used primarily in the treatment of acute lymphoblastic leukemia has the strongest association with increased thrombotic risk. During induction therapy, the incidence of VTE related to L‐asparaginase is approximately 7%–14% [41].

#### 3.7.5 RAMs

Currently, no validated and widely accepted RAM is available for predicting thrombosis in patients with AL [39].

## 4 T‐Cell Engager Therapies as Risk Factors for Venous Thromboembolism

### 4.1 CAR‐T

#### 4.1.1 Epidemiology

Early clinical trials reported very low rates of thrombotic complications. Nevertheless, recent real‐world studies suggest that VTE may occur more frequently in routine clinical practice, particularly during the early post‐infusion period. This discrepancy may be partly explained by potential underreporting of thrombotic events in registrative clinical trials. Available evidence remains limited and heterogeneous, and the true incidence of CAR‐T‐associated thrombosis has not yet been clearly established [43, 44, 45].

#### 4.1.2 Patient‐Related Risk Factors

They have not been well characterized. Therefore, general patient‐related risk factors should be considered, including advanced age, relevant comorbidities, prior history of VTE, reduced performance status, and prolonged hospitalization.

#### 4.1.3 Disease‐Related Risk Factors

They vary according to the underlying indication for CAR‐T therapy (ALL, MM, and NHL). Patients receiving CAR‐T in later lines of therapy and with higher disease burden are at increased thrombotic risk, reflecting more aggressive disease biology and greater systemic inflammation, as well as a higher risk of CAR‐T‐related toxicities, including cytokine release syndrome (CRS) and immune effector cell‐associated neurotoxicity syndrome (ICANS) [46].

#### 4.1.4 Treatment‐Related Risk Factors

To date, no CAR‐T product‐specific differences in thrombotic risk have been demonstrated, including between constructs employing different co‐stimulatory domains (CD28 vs. 4‐1BB), when accounting for the underlying disease indication. Severe CRS and ICANS have been associated with an increased risk of VTE in retrospective studies [47, 48].

##### 4.1.4.1 RAMs

Currently, there is no validated RAM to evaluate thrombotic risk in patients receiving CAR‐T therapy.

### 4.2 Bispecific Antibodies

Bispecific antibodies do not appear to be associated with a clear increased risk of VTE. In randomized clinical trials evaluating their use in ALL, NHL, and MM, VTE events have been rare, with only isolated cases reported for blinatumomab for ALL and epcoritamab for DLBCL [49, 50, 51].

## 5 Additional Risk Factors for CAT Across Hematologic Malignancies

### 5.1 Central Venous Access Devices

The use of CVADs is essential in oncohematologic patients to deliver intravenous medications and minimize treatment‐induced vascular damage. However, their use is associated with an increased thrombotic and infectious risk. The type of catheter is one of the most important risk factors for catheter‐related thrombosis (CRT). The risk is highest with PICCs and lowest with totally implantable venous access devices (PORTs). Specifically, 5%–6% of oncohematologic patients with PICCs experience CRT [52, 53]. However, many hematologists prefer PICCs over PORTs due to their lower cost, ease of implantation, and the possibility of prompt removal in case of infection or CRT. Other catheter‐related risk factors that contribute to the risk of CRT include the catheter material, with polyvinyl chloride and polyethylene having a higher thrombotic risk compared to polyurethane or silicone; the size of the PICC, as the catheter‐to‐vein ratio should be about 1:3; and mispositioning [54, 55].

### 5.2 Inherited Thrombophilia

Inherited thrombophilia refers to a genetic predisposition to VTE. The most well‐established heritable thrombophilias include the factor V Leiden (FVL) variant, the prothrombin gene mutation (FII G20210A), and deficiencies of protein C (PC), protein S (PS), and antithrombin (AT). The contribution of these inherited factors to thrombotic risk in the context of hematologic malignancies remains a subject of ongoing debate. While some studies have reported no association, others have demonstrated up to a sevenfold increase in the risk of CAT in individuals with inherited thrombophilia [56].

Current clinical guidelines do not recommend routine screening for inherited thrombophilia in patients with hematologic malignancies [57, 58, 59, 60].

## 6 DOACs Use in Primary Prophylaxis of VTE

Primary thromboprophylaxis is not routinely recommended for all patients with cancer. It is reserved for individuals classified as having an intermediate or high risk of VTE, based on cancer type or validated risk assessment models, and only in the absence of contraindications to anticoagulation [47, 48]. However, in clinical practice, the systematic adoption of these risk assessment models is not always customary. For example, in patients with lymphoma presenting with bulky disease or venous compression, primary thromboprophylaxis is frequently prescribed, even in individuals who are not classified as intermediate‐to‐high risk according to current validated models. Currently, the standard prophylactic regimen for such patients is LMWH, as DOACs have not yet been approved in most countries for primary prophylaxis in this context [57, 58, 59, 60, 61].

The two major randomized, double‐blind, placebo‐controlled trials, CASSINI and AVERT, have evaluated the role of DOACs in the primary prevention of VTE in cancer patients. Both trials enrolled patients with a Khorana score > 2 and compared DOACs to placebo in a 1:1 ratio. The CASSINI trial assessed rivaroxaban 10 mg once daily, while the AVERT trial evaluated apixaban 2.5 mg twice daily (BID) [62, 63]. The CASSINI trial included 841 participants, among whom 59 (7%) had HL/NHL. The study demonstrated a reduction in VTE incidence with rivaroxaban compared to placebo (2.6% vs. 6.4%) without a significant increase in bleeding risk [62]. In the AVERT trial, 574 patients were enrolled, including 145 with HL/NHL (25.2%) and 15 with MM (2.6%). Apixaban was associated with a lower VTE incidence compared to placebo (4.2% vs. 10.2%), though it was also linked to a higher rate of major bleeding events (3.5% vs. 1.8%) [63].

Thus, several studies have been conducted in oncohematologic patients.

In MM, DOACs have been employed as primary prophylaxis in patients receiving IMiD‐based treatments, primarily evaluated in single‐arm prospective trials or retrospective studies. These reports indicate a VTE incidence ranging from 0% to 5%, with major bleeding rates between 0% and 1.4%. However, the follow‐up periods in these studies have generally been short, typically around 6 months. While these findings are encouraging, further evidence is necessary, particularly from large prospective trials directly comparing DOACs to LMWH [64, 65, 66, 67].

In the context of MPNs, primary thromboprophylaxis typically involves aspirin and cytoreductive therapy. Nonetheless, an ongoing trial, AVAJAK, following the feasibility trial AIRPORT‐MPN, is investigating the role of DOACs for primary thromboprophylaxis in this setting; however, no preliminary results have yet been reported [68, 69].

Recently, the PREVAPIX‐ALL trial evaluated primary thromboprophylaxis with a weight‐adjusted dose of apixaban in an ALL‐pediatric cohort. This phase 3, prospective, randomized study compared apixaban to placebo during induction treatment. Although no statistically significant benefit was observed with apixaban, the safety profile was favorable [70].

In conclusion, more robust evidence supports the use of DOACs for primary thromboprophylaxis in lymphomas and MM. Nonetheless, large, prospective, randomized clinical trials focused on single diseases are required to fully assess the potential utility of DOACs in these settings.

Table 3 summarizes the key evidence on the use of DOACs for primary thromboprophylaxis in patients with hematologic malignancies.

**TABLE 3:** Key evidence on the use of DOACs for primary thromboprophylaxis in patients with hematologic malignancies.

Author (year)	Study design	Patients (*N*)	Oncohematologic patients (*N*, %)	Anticoagulant therapy (*N*, %)	Anticancer‐treatment (*N*, %)	Median follow‐up (days, range)	Major B‐AEs (%)^a^	T‐AEs (%)^a^
Khorana, 2019 [62]^b^	Prospective, randomized, double‐blind	841	HL/NHL (59, 7)	Placebo (26, 44.1) vs. Rivaroxaban 10 mg/daily (33, 55.9)	Not otherwise specified treatment (841, 100)	180	1 vs. 2	6.4 vs. 2.6
Carrier, 2019 [63]^b^	Prospective, randomized, double‐blind	574	HL/NHL (145, 25.2) MM (15, 2.6)	Placebo (283, 49.3) vs. Apixaban 2.5 mg BID (291, 50.7)	Non otherwise specified treatment (574, 100)	180	1.8 vs. 3.5	10.2 vs. 4.2
Pegourie, 2019 [64]	Prospective, single arm	104	MM (104, 100)	Apixaban 2.5 mg BID (104, 100)	IMiDs‐based (104, 100)	210	0.9	1.9
Storrar, 2019 [65]	Retrospective	70	MM (70, 100)	Apixaban 2.5 mg BID (70, 100)	IMiDs‐based (70, 100)	180	1.4	2.9
Cornell, 2020 [66]	Prospective, single‐arm	50	MM (50, 100)	Apixaban 2.5 mg BID (50, 100)	IMiDs‐based (50, 100)	180	0	0
Piedra, 2023 [67]	Retrospective	305	MM (305, 100)	Aspirin 81 mg/daily (223, 73.1) Rivaroxaban 10 mg/daily (82, 26.9)	IMiDs‐based (305, 100)	Non specified (approximately 6–7 months)	0	9.9 vs. 5
O'Brien, 2024 [70]^c^	Prospective, randomized	512	ALL/LL (512, 100)	No thromboprophylaxis (256, 50) Weight adjusted apixaban (256, 50)	Induction treatment (512, 100)	27 days (26–28)	0.8 vs. 0.8	18 vs. 12

Abbreviations: ALL, acute lymphoblastic leukemia; B‐AEs, bleeding adverse events; BID, *bis in die*; HL, Hodgkin lymphoma; IMiDs, immunomodulants; LL, lymphoblastic lymphoma; MM, multiple myeloma; NHL, non Hodgkin lymphoma; T‐AEs, thrombotic adverse events.

^a^ Values are presented as the rate or number of events in the Comparator Arm versus the DOAC Arm where a comparator arm was present; otherwise, only DOAC data are reported.

^b^ Rates of major B‐AEs and T‐AEs are reported for the entire study cohort.

^c^ Pediatric trial.

## 7 DOACs Use in Treatment and Secondary Prophylaxis of VTE

### 7.1 Randomized Clinical Trials on DOACs Use in the Treatment of Cancer‐Associated VTE

Six randomized clinical trials (RCTs) (HOKUSAI‐VTE, SELECT‐D, CARAVAGGIO, ADAM‐VTE, CASTA‐DIVA, and CANVAS) compared DOACs with LMWH in patients with active cancer and acute VTE, with follow‐up periods ranging from 6 to 12 months. These studies demonstrated that DOACs are non‐inferior to LMWH for the treatment of CAT, although they were associated with a higher incidence of clinically relevant non‐major bleeding (CRNMBs). These findings supported the approval of DOACs use in this setting (Table 4) [2, 3, 4, 5, 8, 9]. However, patients with hematologic malignancies were underrepresented in these trials, comprising 10% or less of the total study populations, likely due to their higher baseline risk of bleeding compared to those with solid tumors, with grade ≥ 2 bleedings occurring in 50%–70% of patients [71, 72].

**TABLE 4:** DOACs registrative trials in cancer‐associated VTE treatment: Hematologic malignancies subgroup data.

Author (year)	Study design	Patients (*N*)	Oncohematologic patients (*N*, %)	Anticoagulant therapy (*N*, %)	Anticancer‐treatment (*N*, %)	Median follow‐up (months)	Major B‐AEs (%)^a^	T‐AEs (%)^a^
Raskob, 2018 [2]	Prospective, randomized	1046	111 (10.6)^b^	Dalteparin 120 UI/Kg BID (523, 50) Edoxaban 60 mg/day (523, 50)	Not otherwise specified treatment (1046, 100)^c^	12	3.6 vs. 1.8	7.2 vs. 3.6
Young, 2018 [3]	Prospective, randomized	406	Lymphoma (23, 5.7) MM (5, 1.2) CML (3, 0.7)	Dalteparin 120 UI/Kg BID (203, 50) Edoxaban 60 mg/day (203, 50)	Not otherwise specified treatment (282, 69.5)^c^	6	0	N.A.
Agnelli, 2020 [4]	Prospective, randomized	1155	85 (7.4)^b^	Dalteparin 120 UI/Kg BID (579, 50.1) Apixaban 5 mg BID (576, 49.9)	Not otherwise specified treatment (1155, 100)^c^	6	0	3.8 vs. 6.1
McBane, 2020 [5]	Prospective, randomized	300	Lymphoma (16, 5.3) MM (7, 2.3) Leukemia (4, 1.3) Non specified (1, 0.3)	Dalteparin 120 UI/Kg BID (150, 50) Apixaban 5 mg BID (150, 50)	Not otherwise specified treatment (218, 72.7)^c^	6	N.A.	N.A.
Planquette, 2022 [8]	Prospective, randomized	158	MM/Lymphoma (13, 8.2)	Dalteparin 120 UI/Kg BID (84, 53.2) Rivaroxaban 20 mg/day (74, 46.8)	Not otherwise specified treatment (104, 65.8)^c^	6	N.A.	N.A.
Schrag D., 2023 [9]	Prospective, randomized	638	Lymphoma/MM/CLL (50, 7.8)	LMWH/Fondaparinux (308, 48.3) Investigator's choice DOAC (330, 51.7)	N.A.	6	N.A.	N.A.

Abbreviations: B‐AEs, bleeding adverse events; BID, *bis in die*; CLL, chronic lymphocytic leukemia; CML, chronic myeloid leukemia; LMWH, low molecular weight heparin; MM, multiple myeloma; N.A., not available; T‐AEs, thrombotic adverse events.

^a^ In this table rates of major B‐AEs and T‐AEs are reported for the oncohematologic patients only. Values are presented as the rate or number of events in the Comparator Arm versus the DOAC Arm.

^b^ Not available data stratified *per* disease.

^c^ Not available data stratified per disease.

### 7.2 Randomized Clinical Trials on DOACs Use in the Secondary Prophylaxis of Cancer‐Associated VTE

Two RCTs, AMPLIFY‐EXT and EINSTEIN CHOICE, supported the approval of apixaban 2.5 mg BID and rivaroxaban 10 mg/day for secondary prophylaxis of VTE in cancer patients. However, it is important to note that the AMPLIFY‐EXT trial did not include patients with cancer, while the EINSTEIN CHOICE trial enrolled only three cancer patients, none of whom had hematologic malignancies [6, 7]. Only recently, Mahe et al. [73] published the results of the API‐CAT trial, a prospective, randomized clinical study evaluating the efficacy and safety of apixaban 5 mg twice daily versus 2.5 mg twice daily for secondary prophylaxis of VTE in cancer patients. A total of 1766 patients were enrolled and randomized in a 1:1 ratio, including 135 individuals (7.6%) with oncohematologic malignancies (70 with MM, 27 with NHL, 19 with chronic lymphocytic leukemia (CLL), 2 with HL, 2 with CML, and 15 with other hematologic diseases). Over a median treatment duration of 11.8 months (range: 8.3–12.1 months), VTE recurrence was observed in 2.1% of patients in the low‐dose group compared to 2.8% in the full‐dose group. Major or clinically relevant non‐major bleeding events occurred in 12.1% of patients receiving the low dose and 15.6% of those receiving the full dose. These findings demonstrate the non‐inferiority of the lower apixaban dose compared to the standard dose in this patient population.

### 7.3 Data on DOACs Use in the Treatment and Secondary Prophylaxis of VTE in Hematologic Malignancies

Specific data on oncohematologic diseases are limited and primarily derived from retrospective studies (Table 5) [74, 75, 76, 77, 78, 79, 80, 81, 82, 83]. These studies often include heterogeneous patient populations with various hematologic malignancies and different indications for anticoagulation, with some also encompassing patients treated for arterial thromboembolism (ATE). Overall, the available evidence suggests that DOACs are associated with low rates of recurrent thrombotic events and acceptable bleeding rates in patients with hematologic malignancies, although comparisons across studies are limited by differences in study design and patient characteristics.

**TABLE 5:** Real‐world evidence on DOAC use for VTE treatment, secondary prophylaxis, and AF in patients with hematologic malignancies.

Author (year)	Study design	Patients (*N*)	Disease (*N*, %)	Reason for anticoagulation (*N*, %)	Anticoagulant Therapy (*N*, %)	Anticancer‐treatment (*N*, %)	Median follow‐up (months, range)	Major B‐AEs (%)^a^	T‐AEs (%)^a^
Serrao, 2020 [74]	Retrospective	135	Ph‐negative MPN (50, 37) NHL/HL (27, 20) CLL (14, 10.4) MDS (13, 9.6) MM (11, 8.1) AL (11, 8.1) CML (9, 6.7)	AF (71, 52.6) Acute VTE (46, 34.1) Secondary VTE prophylaxis (18, 13.3)	Rivaroxaban (44, 33) Edoxaban (44, 33) Apixaban (37, 27) Dabigatran (10, 7)	Cytoreductive agents (41, 30.4) TKI (23, 17) iCHT (23, 17) IMIDs (7, 5.2) Venetoclax (3, 2.2)	18 (4–73)	0	0
Kim, 2020 [75]	Retrospective	103	NHL/HL (40, 38.9) MM (26, 25.2) AL (18, 17.5) CLL (13, 12.6) MDS (2, 1.9) Other (3, 2.9) CML (1, 1)	VTE (90, 87.4) AF (13, 12.6)	Rivaroxaban (90, 87.4) Dabigatran (8, 7.8) Apixaban (5, 4.9)	N.A.	50.5 (0.1–57.9)	3.9	1.9
Huenerbein, 2020 [76]	Retrospective	71	PV (30, 42.3) PMF (20, 28.2) ET (19, 26.8) MPN‐U (2, 2.8)	VTE (62, 87.3) ATE (9, 12.7)	VKA (45, 63.4) Rivaroxaban (21, 29.6) Apixaban (5, 7)	Cytoreductive agents (39, 63.9)	18 (1–102)	8.9 vs. 7.7	48.8 vs. 15.4
Fedorov, 2021 [77]	Retrospective	30	ET (23, 76.7) PV (6, 20) MPN‐U (1, 3.3)	VTE (20, 66.7) ATE (8, 26.7) ATE+VTE (2, 6.7)	VKA (11, 36.7) DOACs (19, 63.3)	Cytoreductive agents (22, 73.3)	8.6 (4.4–21.1)	0	9.1 vs. 5.3
Barbui, 2021 [78]	Retrospective	442	PV (178, 40) ET (172, 39) PMF (92, 21)	Secondary VTE prophylaxis (239, 54.1) AF (203, 45.9)	Rivaroxaban (187, 42.3) Apixaban (157, 35.5) Dabigatran (50, 11.3) Edoxaban (48, 10.9)	Cytoreductive agents (398, 90)	> 12 (N.A.)	5.9	7.2
How, 2021 [79]	Retrospective	133	PV (76, 57.1) ET (35, 26.3) MPN‐U (11, 8.3) Post‐ET/PV MF (7, 5.3) PMF (4, 3)	VTE (75, 56.4) AF (46, 34.6) ATE (12, 9)	Apixaban (83, 62.4) Rivaroxaban (42, 31.6) Dabigatran (7, 5.3) Edoxaban (1, 0.8)	Cytoreductive agents (110, 82)	37	5.3	12
Herbreteau, 2022 [80]	Retrospective	135	PV (59, 43.7) ET (52, 38.5) Post‐ET/PV MF (10, 7.4) MPN‐U (9, 6.7) PMF (4, 3) Pre‐PMF (1, 0.7)	AF (66, 48.9) VTE (63, 46.7) ATE (5, 3.7) Other (1, 0.7)	Apixaban (68, 50.4) Rivaroxaban (67, 49.6)	Cytoreductive agents (90, 66.7)	23.8	5.9	14.8
Chistolini, 2023 [81]	Retrospective	53	NHL/HL (26, 49) MPN (16, 30.3) MM (7, 13.1) CLL (3, 5.6) HCL (1, 1.8)	Secondary VTE prophylaxis (53, 10)	Rivaroxaban 10 mg/day (36, 67.9) Apixaban 2.5 mg BID (17, 32.1)	N.A.	13.3 (5.6–45.7)	1.9	0
Robinson, 2023 [82]	Retrospective	143	LNH/HL (83, 58) MM (32, 22.4) Ph‐negative MPN (21, 14.7) AL 5, (3.5) CML (2, 1.4)	Acute VTE (143, 100)	LMWH (96, 67.1) Apixaban (27, 18.9) Rivaroxaban (20, 14)	N.A.	9.6 (3–12)	9.4 vs. 2.1	0 vs. 4.3
Biglietto, 2025 [83]	Retrospective	228	JAK2^+^‐MPN (70, 30.7) NHL/HL (69, 30.3) CLL (21, 9.2) MM (21, 9.2) JAK2^−^‐MPN (17, 7.5) AL (11, 4.8) MDS (10, 4.4) PNH (7, 3.1) AA (1, 0.4) Others (1, 0.4)	VTE (164, 71.9) AF (64, 28.1)	Apixaban (106, 46.5) Edoxaban (63, 28.1) Rivaroxaban (53, 23.2) Dabigatran (6, 2.6)	CHT ± RT (57, 25) Immunotherapy (77, 33.8) iCHR ± RT (23, 10.1)	34 (3.7–113.5)	1.3	3.9

Abbreviations: AA, aplastic anemia; AF, atrial fibrillation; AL, acute leukemia; ATE, arterial thromboembolism; B‐AEs, bleeding adverse events; BID, *bis in die*; CLL, chronic lymphocytic leukemia; CML, chronic myeloid leukemia; ET, essential thrombocythemia; HCL, hairy cell leukemia; HL, Hodgkin lymphoma; iCHT, immuno‐chemotherapy; IMIDs, immunomodulants; LMWH, low molecular weight heparin; MDS, myelodysplastic neoplasm; MM, multiple myeloma; MPN‐U, unclassifiable myeloproliferative neoplasm; N.A., not available; NHL, non Hodgkin lymphoma; Ph‐negative MPN, Philadelphia negative myeloproliferative neoplasm; PMF, primary myelofibrosis; PNH, paroxysmal nocturnal hemoglobinuria; pre‐PMF, prefibrotic myelofibrosis; PV, polycythemia vera; RT, radiotherapy; T‐AEs, thrombotic adverse events; TKI, tyrosine kinase inhibitors; VKA, vitamin K inhibitor; VTE, venous thromboembolism.

^a^ Values are presented as the rate or number of events in the Comparator Arm versus the DOAC Arm where a comparator arm was present; otherwise, only DOAC data are reported.

Regarding acute VTE treatment, two recent retrospective analyses offer a comparison between LMWH and DOACs. Kim et al. [75] reported a higher incidence of CRNMB with DOACs (28.2%), along with a similar incidence of VTE or stroke compared with LMWH; however, most patients in their cohort had undergone hematopoietic stem cell transplantation and therefore had a high baseline risk for both thrombotic and hemorrhagic events. Conversely, Robinson et al. [82] reported a higher bleeding rate with LMWH than with DOACs (9.4% vs. 2.1%); however, this may reflect confounding by indication, as LMWH might have been preferentially prescribed to patients with an intrinsically higher bleeding risk. A recent retrospective study [76] comparing VKAs and DOACs for ATE and VTE in 71 patients with Ph‐negative MPNs reported a higher rate of thrombotic recurrence with VKAs and a similar rate of bleeding events. These findings are supported by a meta‐analysis [77] conducted in the same setting, which showed no significant differences in bleeding or thrombosis recurrence between VKAs and DOACs; however, only 30 patients were included. Two studies [79, 80] evaluating DOACs in patients with Ph‐negative MPNs for various indications, including acute VTE treatment, reported higher bleeding rates among those receiving concomitant low‐dose aspirin.

For secondary VTE prophylaxis, two retrospective analyses provide the main sources of evidence. Barbui et al. [78] described their experience with DOACs in 442 patients with Ph‐negative MPNs treated for secondary VTE prophylaxis or atrial fibrillation (AF). The study reported an incidence rate of thrombosis of 4.5 per 100 patient‐years in VTE patients and 2.1 per 100 patient‐years in AF patients. The incidence of major bleeding was 2.3 and 3.0 per 100 patient‐years in patients with AF and VTE, respectively. Chistolini et al. [81] described their experience with DOACs for secondary VTE prophylaxis in a heterogeneous cohort of 53 onco‐hematologic patients compared with non‐neoplastic patients, reporting no thrombotic complications and a low incidence of bleeding (5.1 events per 100 patient‐years).

### 7.4 DOACs Use in Catheter Related Thrombosis

Specific data on oncohematologic malignancies is almost nonexistent. Current practice relies heavily on studies about the management of CRT in solid cancers, where DOACs have demonstrated favorable safety and efficacy profiles. Dedicated prospective clinical trials focusing on oncohematologic patients are critically needed [55, 84].

## 8 DOACs Laboratory Monitoring

Due to their favorable pharmacokinetic profile, DOACs plasma levels were not monitored in RCTs, and such monitoring is not routinely indicated in clinical practice. Nevertheless, it may be potentially useful in specific clinical scenarios, including recurrent VTE, bleeding complications during DOAC therapy, non‐elective surgical procedures, assessment of pharmacological interactions, and suspected drug malabsorption [85, 86, 87].

This form of monitoring may be particularly valuable in the oncohematologic setting, primarily due to the potential for pharmacological interactions. All DOACs are substrates of P‐glycoprotein and CYP3A4. Several agents used in the treatment of oncohematologic malignancies act as inducers or inhibitors of these metabolic pathways (Table 6) [87].

**TABLE 6:** Principal potential interactions between anticancer drugs and DOACs in hematologic malignancies (adapted from Ferri et al. [87]).

Drug	Effect on P‐gp and CYP	Dabigatran	Rivaroxaban	Apixaban	Edoxaban
Vinblastine, Vincristine	CYP3A4/P‐gp competition	Mild decrease in AUC predicted			
Doxorubicin	CYP3A4/P‐gp competition	Decrease in AUC predicted			
Dexamethasone	Strong CYP3A4/P‐gp induction; CYP3A4/P‐gp competition	Possible decrease in AUC predicted			
Tacrolimus	Strong to moderate P‐gp inhibition; moderate CYP3A4 inhibition; CYP3A4/P‐gp competition	Strong increase of AUC predicted	Possible increase in AUC predicted	Possible increase in AUC predicted	Moderate increase in AUC predicted
Cyclosporine	Strong to moderate P‐gp inhibition; moderate CYP3A4 inhibition; CYP3A4/P‐gp competition	Strong increase of AUC predicted	+46% AUC, + 2 fold Cmax	+20% AUC, +40% Cmax	+73% AUC (reduce to 30 mg as indicated by label)
Imatinib, Crizotinib	Strong P‐gp inhibition, moderate CYP3A4 inhibition; CYP3A4/P‐gp competition	Strong increase in AUC predicted			
Dasatinib	Mild CYP3A4 inhibition; CYP3A4/P‐gp competition	Possible increase in AUC predicted			
Nilotinib	Moderate‐to‐strong P‐gp inhibition; mild CYP3A4 inhibition; CYP3A4/P‐gp competition	Possible increase in AUC predicted			
Sunitinib, Avapritinib, Carfilzomib, Gladesgib, Ponatinib	P‐gp inhibition; CYP3A4 competition	Possible increase in AUC predicted			
Asciminib	P‐gp inhibition; Moderate CYP3A4 inhibition; CYP3A4/P‐gp competition	Possible increase in AUC predicted			
Gilteritinib, Pemigatinib	P‐gp inhibition	Possible increase in AUC predicted			
Vemurafenib	P‐gp inhibition; Moderate CYP3A4 induction	Moderate increase in AUC predicted	Possible variation in AUC predicted		
Ruxolitinib	P‐gp inhibition; CYP3A4 competition	Possible increase in AUC predicted			
Fedratinib	P‐gp inhibition; CYP3A4 inhibition	Possible increase in AUC predicted			
Ibrutinib	P‐gp inhibition; CYP3A4 competition	Possible increase in AUC predicted			
Acalabrutinib, Zanubrutinib	CYP3A4 and P‐gp competition	Possible increase in AUC predicted			
Venetoclax	P‐gp inhibition; CYP3A4 and P‐gp competition	Possible increase in AUC predicted			

Abbreviations: AUC, area under the curve (DOAC concentration); Cmax, maximum concentration; CYP, cytochrome P 450; P‐gp, P‐glycoprotein.

Recently, two studies have addressed this issue within the oncohematologic context. In the study by Serrao et al. [86], DOAC plasma levels were measured at the time of bleeding events, revealing elevated concentrations in two patients receiving ibrutinib and edoxaban, one patient receiving imatinib and edoxaban, and one patient receiving nilotinib and rivaroxaban. Conversely, the study by Punnachet et al. [88] demonstrated that R‐CHOP chemotherapy does not significantly affect DOAC plasma levels.

These findings suggest that laboratory monitoring of DOACs may be beneficial in the oncohematologic setting, particularly in the context of potential drug–drug interactions; however, further data and standardization of the anti‐Xa and the anti‐IIa assays are required to support routine implementation.

## 9 Clinical Scenarios Requiring Specific Anticoagulation Considerations

Certain clinical scenarios deserve special consideration because of their unique thrombotic and hemorrhagic profiles and the limited evidence available regarding DOAC use. These include APL‐associated coagulopathy, thrombosis at unusual sites, and VTE occurring after T‐cell engager therapies.

### 9.1 Acute Promyelocytic Leukemia

APL is characterized by a thrombotic–hemorrhagic coagulopathy resulting from simultaneous activation of coagulation and fibrinolysis. This complex hemostatic imbalance leads to a substantial risk of both bleeding and thrombosis and contributes to early mortality. In the ATRA/ATO era, hemorrhagic complications remain the predominant clinical manifestation, occurring in 76%–89% of patients, whereas thrombotic events have been reported in 5.1%–20.6% of cases [42]. Effective treatment of APL with ATRA combined with ATO or chemotherapy, along with appropriate supportive measures (maintaining functional fibrinogen levels > 100–150 mg/dL, INR < 1.5, and platelet counts > 30 × 10^9^/L), has significantly reduced the incidence of these complications.

When thrombosis occurs, management should be tailored to the single patient, as evidence regarding thromboembolism management in APL remains limited. Current guidelines recommend subcutaneous LMWH or, in cases of severe thrombosis, intravenous unfractionated heparin (UFH), with dose adjustments based on platelet count [89]. Data regarding DOAC use in APL are currently lacking, and their role remains undefined, particularly during active disease phases characterized by coagulopathy and high bleeding risk.

During ATRA/ATO‐related differentiation syndrome, anticoagulation management represents an additional challenge. The inflammatory response, endothelial activation, leukocytosis, and increased vascular permeability associated with this condition may further promote a prothrombotic state; however, concomitant thrombocytopenia and persistent coagulation abnormalities frequently limit the safety of anticoagulant therapy. In the absence of prospective evidence, anticoagulation decisions should be individualized according to the clinical scenario, considering the severity of thrombosis, platelet count, bleeding risk, and severity of the eventual differentiation syndrome. Dose reduction or temporary interruption of anticoagulation may be required in patients with significant bleeding risk or severe coagulopathy [42, 89].

### 9.2 Splanchnic and Cerebral Vein Thrombosis

Splanchnic (SVT) and cerebral (CVT) vein thromboses in onco‐hematologic patients are strongly associated with Ph‐negative MPNs [90, 91]. SVT, including Budd–Chiari syndrome, extrahepatic portal vein obstruction, and mesenteric vein thrombosis, occurs in approximately 1%–10% of Ph‐negative MPNs patients [90].

CVT is less frequent, occurring in fewer than 1% of patients with Ph‐negative MPNs; however, it should not be overlooked, as approximately 3.8% of patients presenting with CVT are found to have an underlying Ph‐negative MPN [91].

Evidence regarding DOACs use in Ph‐negative MPN‐associated SVT remains limited and is mainly derived from observational studies. In a large retrospective cohort of 181 patients with Ph‐negative MPN‐associated SVT treated mostly with VKA, De Stefano et al. highlighted the high risk of recurrent thrombotic events in this population, supporting the importance of long‐term anticoagulation strategies; furthermore, there was a high incidence of major bleeding in patients receiving VKA (2 per 100 patient‐years) [90]. More recently, Ageno et al. conducted a prospective single‐arm study evaluating rivaroxaban in 100 patients with non‐cirrhotic SVT, among whom 13 of the 50 tested patients were positive for the JAK2 V617F mutation. Rivaroxaban was associated with low rates of recurrent SVT (2.1%) and major bleeding (2.1%) at 3 months [92].

Data regarding DOACs use in CVT in Ph‐negative‐MPN remain limited, and therapeutic decisions are generally extrapolated from studies in CVT and other unusual‐site thromboses in the general population, as disease‐specific evidence is currently lacking.

Further prospective randomized studies specifically evaluating the optimal anticoagulation strategy in this setting are warranted.

### 9.3 Venous Thromboembolism Following T‐Cell Engager Therapies

The management of VTE in patients treated with T‐cell engager therapies, including CAR‐T cells and bispecific antibodies, is complex due to CRS, ICANS, and treatment‐related cytopenias. In this setting, anticoagulation strategies should be individualized and adapted to the evolving clinical scenario.

In patients receiving CAR‐T cell therapy, parenteral anticoagulation with LMWH should be favored over DOACs during the early post‐infusion period, which is characterized by fluctuating clinical status and frequent cytopenias. Once clinical stability and adequate platelet recovery are achieved, a transition to DOACs may be considered in selected patients [93]. Similar principles apply to patients receiving bispecific antibodies; however, a substantial lack of evidence persists for both therapeutic modalities, underscoring the need for real‐world registries to guide clinical practice.

### 9.4 Thrombocytopenia

Thrombocytopenia is a common complication in cancer patients, particularly in hematologic malignancies, and presents significant challenges in the management of CAT due to an elevated risk of bleeding [71, 94]. Current clinical guidelines recommend the use of DOACs in patients with a platelet count above 50 × 10^9^/L [57, 58, 59, 60, 61, 94]. However, pivotal clinical trials investigating DOACs for CAT management [2, 3, 4, 5, 8, 9] included only a small subset of patients with platelet counts between 50 × 10^9^/L and 100 × 10^9^/L. Preliminary data on the use of DOACs in this challenging population have emerged from small, single‐center experiences, like that from Abbas et al. [95]. They observed comparable rates of thrombotic recurrence and major bleeding between DOACs and LMWH in patients with CAT within this platelet range, although the follow‐up period was limited. Nevertheless, oncohematologic patients with thrombocytopenia and CAT face an intrinsically high risk of both VTE recurrence and bleeding, irrespective of the anticoagulation strategy [96].

In this context, the management of the underlying thrombocytopenia is essential. The recent ISTH guidance [94] provides a comprehensive framework for addressing chemotherapy‐induced thrombocytopenia (CIT), emphasizing that optimizing platelet support is a key component of the global care of these patients.

The lack of data regarding this population highlights the need for prospective trials to clarify the safety and efficacy of anticoagulation strategies in thrombocytopenic patients with CAT.

### 9.5 Heparin‐Induced Thrombocytopenia

Heparin‐induced thrombocytopenia (HIT) is an uncommon but clinically important complication of UFH and LMWH therapy. Oncohematologic patient‐specific guidance for the management of HIT in this setting is currently lacking. In this population, HIT should be managed according to established guidelines for the general population, including immediate discontinuation of heparin products and initiation of an alternative non‐heparin anticoagulant, such as argatroban, bivalirudin, fondaparinux, or DOACs. Evidence supporting DOACs use remains limited and is primarily derived from observational studies, with the most data available for rivaroxaban [97, 98].

## 10 Proposed Algorithm for DOAC Use in Treatment and Secondary Prophylaxis of VTE in Oncohematologic Patients

Given the complexity of managing VTE in hematologic malignancies, we propose a clinical algorithm (Figure 2) to guide the selection of anticoagulant therapy. The decision‐making process begins with a baseline assessment of the patient's bleeding risk, with a primary focus on the platelet count. According to current consensus, a threshold of 50 × 10^9^/L is recommended for the safe initiation of DOACs. Subsequently, a rigorous screening for potential drug–drug interactions is crucial, particularly evaluating the co‐administration of CYP3A4 or P‐gp inhibitors/inducers. In cases where the platelet count is < 50 × 10^9^/L or significant drug–drug interactions occur, LMWH remains the standard of care.

**FIGURE 2:**
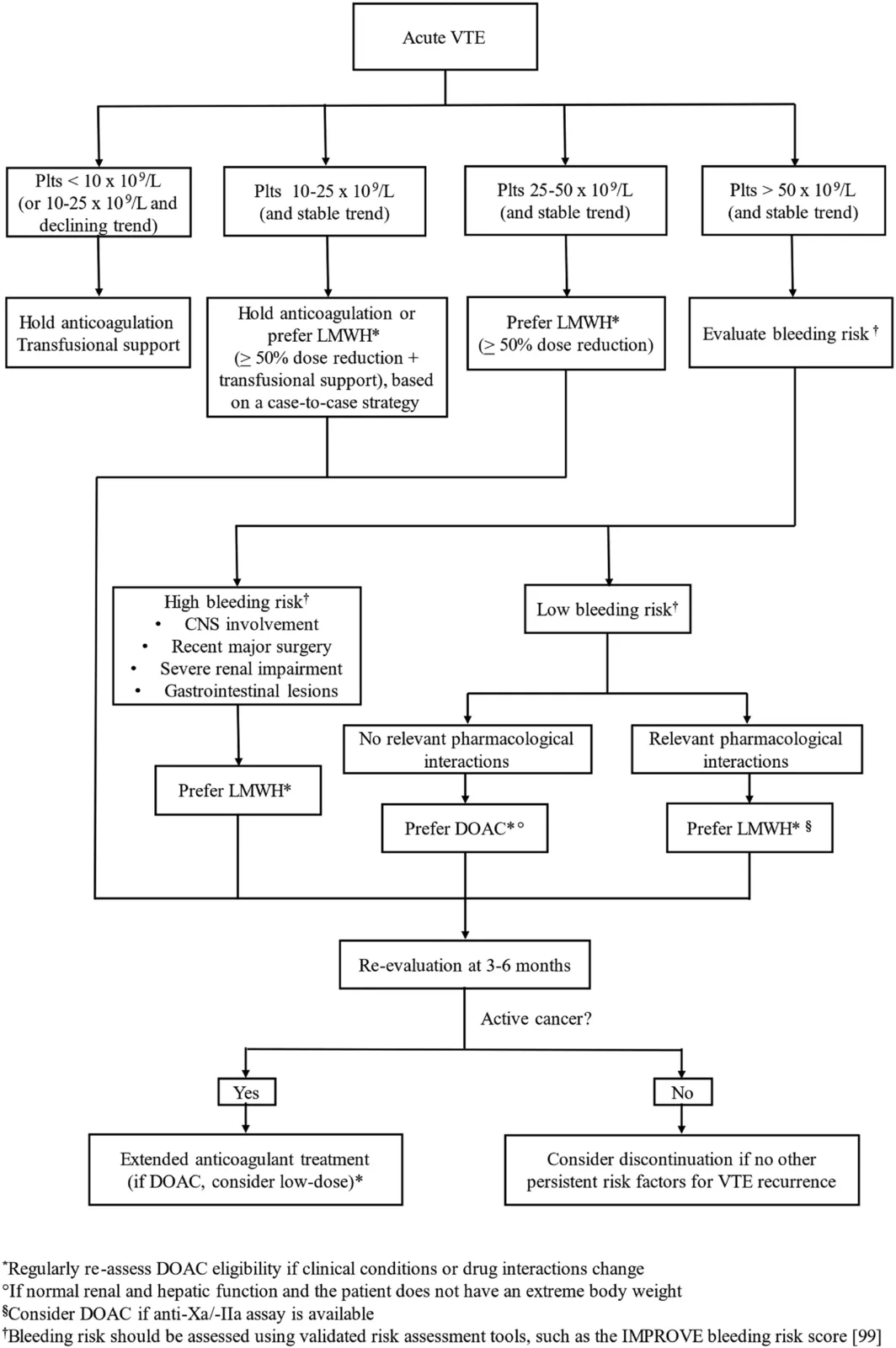
Proposed algorithm for DOAC use in treatment and secondary prophylaxis of VTE in hematologic malignancies.

## 11 Conclusions

Oncohematologic patients face a significantly elevated risk of both bleeding and thromboembolic complications, presenting complex challenges in anticoagulation management. Existing risk assessment tools for thrombosis in this population are suboptimal and require refinement to better guide clinical decision‐making. DOACs are a valid therapeutic option for the treatment and secondary prevention of VTE in oncohematologic settings. In patients receiving potentially interacting anticancer therapies, laboratory monitoring of DOACs levels may be beneficial for optimizing treatment safety and efficacy. Nonetheless, thrombocytopenia remains a significant limitation to the use of DOACs, as their safety and efficacy are not well established in patients with platelet counts between 50 × 10^9^/L and 100 × 10^9^/L. Moreover, current clinical guidelines contraindicate the use of DOACs in individuals with platelet counts below 50 × 10^9^/L. Finally, there is a pressing need for prospective clinical trials focused on specific hematologic malignancies to more precisely evaluate the safety, efficacy, and appropriate use of DOACs in these diverse and high‐risk patient populations.

## Author Contributions

Antonio Chistolini, Mario Biglietto: writing – review and editing, writing – original draft, methodology, conceptualization. Massimo Breccia, Cristina Santoro, Erminia Baldacci: writing – review and editing, methodology, conceptualization. Silvia Sorella, Sabina Chiaretti, Marcello Capriata, Livia Donzelli, Gianna Maria D'Elia, Salvatore Giacomo Morano, Ilaria Del Giudice, Maurizio Martelli: writing – review and editing, methodology.

## Funding

The authors have nothing to report.

## Conflicts of Interest

The authors declare no conflicts of interest.

## Data Availability

Data sharing not applicable to this article as no datasets were generated or analysed during the current study.
